# Carbon allocation and competition maintain variation in plant root mutualisms

**DOI:** 10.1002/ece3.4118

**Published:** 2018-05-04

**Authors:** Natalie Christian, James D. Bever

**Affiliations:** ^1^ Evolution, Ecology and Behavior Program Department of Biology Indiana University Bloomington Indiana; ^2^ Department of Ecology and Evolutionary Biology The University of Kansas Lawrence Kansas

**Keywords:** biological market theory, cheating, preferential allocation, resource competition, species coexistence

## Abstract

Plants engage in multiple root symbioses that offer varying degrees of benefit. We asked how variation in partner quality persists using a resource‐ratio model of population growth. We considered the plant's ability to preferentially allocate carbon to mutualists and competition for plant carbon between mutualist and nonmutualist symbionts. We treated carbon as two nutritionally interchangeable, but temporally separated, resources—carbon allocated indiscriminately for the construction of the symbiosis, and carbon preferentially allocated to the mutualist after symbiosis establishment and assessment. This approach demonstrated that coexistence of mutualists and nonmutualists is possible when fidelity of the plant to the mutualist and the cost of mutualism mediate resource competition. Furthermore, it allowed us to trace symbiont population dynamics given varying degrees of carbon allocation. Specifically, coexistence occurs at intermediate levels of preferential allocation. Our findings are consistent with previous empirical studies as well the application of biological market theory to plantroot symbioses.

## 1 INTRODUCTION

Mutualisms are interactions between species that confer benefit to both partners (Boucher, James, & Keeler, 1982). Although mutualisms are ubiquitous, persistent, and critical, evolutionary theory predicts that in the absence of stabilizing mechanisms (Heath & Tiffin, 2009) or perfectly aligned partner fitness (Friesen, 2012), every reciprocal partnership is susceptible to exploitation (Yu, 2001). If multiple symbionts obtain some good from a host via a mutualistic interaction, selection would favor symbionts that provide reduced benefit in exchange for the good and thus incur less cost while receiving full benefit (Yu, 2001). Such exploitation of the mutualistic system, in which “cheaters” take advantage of the mutualism, should result in its inevitable collapse (Kiers & Denison, 2008). Empirically, however, cheating does not pose a strong threat to the persistence of mutualisms (Jones et al., 2015): Horizontal mutualisms are stable, and cheating does not necessarily lead to the collapse of symbiotic associations. Rather, stable associations of mutualists and cheaters can persist, even over long spans of evolutionary time (Ferriere, Bronstein, Rinaldi, Law, & Gauduchon, 2002; Sachs & Simms, 2006). Cheating has been documented in multiple types of mutualistic systems, such as plant–pollinator interactions (Jandér & Herre, 2010) and plant–microbial mutualisms. For instance, bacterial rhizobia cheaters may form ineffective nodules on plant roots, exploiting plant carbon without providing nitrogen to the plant in return (Kiers, Rousseau, West, & Denison, 2003). Moreover, genotypes of rhizobia that confer less host benefit have also been shown to be more fit than more cooperative genotypes (Porter & Simms, 2014). Mycorrhizal fungi also vary in benefit and may even adopt a conditional parasitic lifestyle, sequestering plant carbon without reciprocating with phosphorous provision (Johnson, Graham, & Smith, 1997). The diversity and frequency of cheating in these systems have led to a classic paradox: How do mutualistic plant–root symbiont interactions persist in the face of exploitation?

A growing body of evidence suggests that plants can preferentially allocate resources to more mutualistic partners (Bever, Richardson, Lawrence, Holmes, & Watson, 2009; Jandér & Herre, 2016; Ji & Bever, 2016; Kiers et al., 2011; Zheng, Ji, Zhang, Zhang, & Bever, 2015). Preferential allocation, like sanctions, encourages honest partners and selects against cheaters (Bever, 2015), but this could potentially erode the variation upon which such selection operates (Foster & Kokko, 2006). Thus, given preferential allocation, why do cheaters not go extinct? This question has prompted a more modern iteration of the paradox of mutualism, asking what maintains variation for partner quality in mutualisms (Foster & Kokko, 2006; Heath & Stinchcombe, 2014). Several solutions to this new paradox have recently been proposed. Friesen and Mathias (2010) showed that higher coinfection rates by symbionts increase the potential for symbiont diversification by increasing social conflict, in which more effective symbionts are exploited by less effective symbionts. Steidinger and Bever (2014) suggested that the cost to discriminate against cheaters generates negative community feedback, promoting hosts that do not discriminate. Moeller and Neubert (2015) found that when environments are variable, plants may invest in low‐quality partners in response to diminishing returns on investments in high‐quality partners, which also maintains cheaters and mutualists. Bever (2015) identified that negative physiological feedbacks for nutritional mutualists allow cheaters and mutualists to coexist. Steidinger and Bever (2016) demonstrated that mixed colonization of modules (e.g., rhizobia nodules) permits coexistence of mutualist and exploiter partners, even with host discrimination. Most recently, Yoder and Tiffin (2017) suggested that incorporating host recognition of symbiont signals alongside host sanctions against ineffective partners maintains variation. Thus, our understanding of potential mechanisms by which variation in partner quality may be maintained in plant–root symbiont mutualisms is rapidly increasing.

In this article, we identify another, and perhaps more common, stabilizing mechanism of root mutualisms: symbiont competition for different types of host carbon. In order to more fully understand the maintenance of partner variation in plantroot symbioses, we must recognize exploitation competition as an important factor in this dynamic (Ferrière, Gauduchon, & Bronstein, 2007; Jones, Bronstein, & Ferrière, 2012). For instance, Ferrière et al. (2007) demonstrated that mutualisms are better able to persist when more beneficial partners enjoy a competitive advantage over more exploitative partners. Moreover, we explore the consequences of the fact that, in the absence of honest indicators of symbiont quality, hosts must initially construct sites of symbiosis and invest in their symbionts prior to assessing their effectiveness (Bever, 2015). Thus, carbon allocation comes in two basic forms: that which is indiscriminately allocated toward root growth and to initiate the establishment and assessment of root symbioses and that which is then preferentially allocated to a mutualist partner. For example, Ji and Bever (2016) estimated that while 25% of total plant fixed carbon is preferentially allocated to a beneficial mycorrhizal fungus in exchange for soil phosphorous, there is low but consistent proportion of fixed carbon that plants indiscriminately allocate to roots and their symbionts that is available to nonbeneficial symbionts, even with spatial structure (Bever et al., 2009). Here, we examine the conditions in which the presence of these two forms of host investment can contribute to symbiont coexistence.

## 2 THE MODEL

We build from the framework of Bever (2015), which predicts the coexistence of a more beneficial and less beneficial root symbiont (designated as the mutualist (*M*) and nonmutualist (*N*), respectively), even in the face of considerable costs to the mutualist of provisioning the host plant with nutrients. In that model, these costs are overcome by incorporating physiological plasticity of plants to preferentially allocate carbon (C) to the mutualist. In essence, the mutualist and the nonmutualist are thus competing for carbon allocated by a shared host plant. Carbon allocation to each competitor depends on the fidelity (*f*) of the plant to the mutualist. Fidelity to the mutualist likely depends upon the spatial structure of the microbial community (greater spatial structure leads to greater fidelity), and the ability of the plant to preferentially allocate resources to more mutualistic associates in the absence of spatial structure. Additionally, fidelity may be a function of the morphological intimacy of the association, such as when the site of nutrient exchange involves the construction of nodules or arbuscules (greater morphological intimacy leads to greater fidelity). Fidelity is a parameter that can range from 0 to 1, where 1 indicates that only the mutualist receives carbon, and 0 indicates that the carbon is randomly distributed and is equally accessible by both mutualistic and nonmutualistic symbionts through scramble competition (Bever, 2015). These and all subsequent model terms are defined in Table 1.

**Table 1:** Description of model terms

Model term	Definition
*W*_*M*_	Growth rate of mutualist symbionts
*W*_*N*_	Growth rate of nonmutualist symbionts
C	Total carbon allocated to symbionts
*f*	Fidelity of plant allocation to mutualist symbionts
*s*	Cost of mutualism
*b*_max_	Maximum growth rate of symbionts
k	Half‐saturation constant
*d*	Constant death rate
C_c_	Construction carbon that is allocated indiscriminately to symbionts to construct the site of symbiosis
C_a_	Allocation carbon that is discriminately and preferentially allocated to mutualist symbionts
C*	Critical amount of carbon that sustains populations above the constant death rate (*d*)
Ĉ$\hat{C}$	Amount of carbon that sustains populations at an equilibrium growth rate

It is assumed that there are energetic costs associated with being a mutualist, which reduce the growth rate of the mutualist relative to that of the nonmutualist (Bennett & Bever, 2009). The costs of provisioning host plants with nutrients are incurred both in the presence and absence of preferential allocation of carbon by the plant. In this model, the cost of mutualism, *s*, can range from 0 (no cost of mutualism) to 1 (complete cost of mutualism) (Bever, 2015).

These parameters were applied to a resource‐ratio (R*) model of population growth. Based on Monod kinetics, this model describes resource‐dependent growth to predict competitive interactions as a function of concentration of resources (Grover, 1997; Monod, 1950; Tilman, 1976). The resource‐ratio model stipulates that there is a specific concentration of resources, denoted as R*, which is the limiting level of a resource needed to keep population birth rate above a constant death rate. If the concentration of resources falls below R*, the population will collapse. In a competitive context, a species with a lower R* can reduce the level of this essential resource below that of its competitor, resulting in the extinction of the latter. In this case, where the resource in question is carbon (C) the growth rates of the nonmutualist and the mutualist, respectively, are

(1) $$W_{N}=\frac{b_{\max}\left(1-f\right)C}{k+(1-f)C}-d$$

and

(2) $$W_{M}=\frac{b_{\max}\left(1-s\right)C}{k+C}-d.$$

Here, *W*_*N*_ and *W*_*M*_ are the per capita growth rates of the nonmutualist and the mutualist, respectively. Per capita growth rate is affected by *b*_max,_ the maximum birth rate of each symbiont, which is reduced in the nonmutualist with increasing fidelity (*f*) of the plant to the mutualist, and is reduced for the mutualist due to costs (*s*) associated with provisioning host plants with nutrients. Additionally, *f* modifies the amount of C to which the nonmutualist has access, and thus occurs in the denominator in addition to the numerator in Equation (1). In accordance with Monod kinetics, *k* is the half‐saturation constant and is always less than *b*_max_. For both the mutualist and nonmutualist, *d* is the constant death rate. The per capita birth rates for the mutualist and the nonmutualist cross (i.e., $W_{M}=W_{N}$) when

(3) $$C=\frac{k\left(s-f\right)}{s\left(f-1\right)}.$$

By plugging Equation (3) into either of the per capita birth functions, we find that the birth rate for the mutualist and nonmutualist at their point of intersection is

(4) $$W=\frac{b_{\max}\left(f-s\right)}{f}.$$

If the constant death rate, *d*, is above this point, then the nonmutualist will always have a competitive advantage and the mutualist will decline to extinction. Mutualism is only possible when *d* is below this point. Rearranging this condition (see Appendix S1), we identify that the persistence of mutualism requires that the fidelity of plant investment,

(5) $$f>\frac{b_{\max}s}{b_{\max}-d}.$$

This condition is more restrictive than in the absence of explicit consideration of resource dynamics (Bever, 2015). Given that $b_{\max}>d$ in order for any fungi to grow, the persistence of mutualism is only possible when $\frac{b_{\max}s}{b_{\max}-d}$ is less than 1, or the cost of mutualism

(6) $$s<\frac{b_{\max-d}}{b_{\max}}.$$

As in previous models, the mutualist and nonmutualist cannot coexist on one carbon source without negative physiological feedback (Bever, 2015) or negative community feedback (Steidinger & Bever, 2014).

We posit that when naïve plant roots first associate with symbionts in the soil, plants must invest in the association by providing some amount of carbon in order for symbionts to enter the root and construct their physical relationship with the plant. This initial deposit of carbon is designated as C_c_ (construction carbon) and is a cost incurred by the plant from which both the mutualist and nonmutualist benefit. Once colonization of plant roots by symbiont associates has occurred, the plant is able to recognize more beneficial partners and preferentially allocates carbon, C_a_ (allocation carbon), to these mutualists. While these two carbon sources are necessarily sequenced with construction preceding allocation, in the current model we represent these allocations as continuous investments, consistent with a growing plant root system. Thus, growth rates for the nonmutualist and mutualist are represented by

(7) $$W_{N}=\frac{b_{\max}C_{N}}{k+C_{N}}-d$$

and

(8) $$W_{M}=\frac{b_{\max}\left(1-s\right)C_{M}}{k+C_{M}}-d,$$

respectively, where $C_{N}=C_{c}+\left(1-f\right)C_{a}$ and $C_{M}=C_{c}+C_{a}$.

Because the density of both the nonmutualist and the mutualist depends on carbon obtained from the plant, there is a limiting level of carbon necessary to sustain the populations. In other words, there is a critical amount of carbon, C*, which keeps births of symbionts above a constant death rate, *d*. Should C* fall below *d*, the population will collapse. The critical levels for both sources of carbon (that used to construct the symbiont–plant mutualism and that used to preferentially reward the mutualist) can be obtained by solving for the equilibrium growth rate of each symbiont, that is, the condition in which $W_{N}=0$ and $W_{M}=0$ (see Appendix S1). Thus, at equilibrium,

(9) $${\hat{C}}_{N}^{*}=\frac{\mathrm{dk}}{b_{\max}-d}$$

and

(10) $${\hat{C}}_{M}^{*}=\frac{\mathrm{dk}}{\left(b_{\max}\right)\left(1-s\right)-d.}$$

Consequently, by assuming that C_a_ and C_c_ are completely interchangeable, and that they comprise 100% of the symbionts' carbon source, it can be determined that for the nonmutualist

(11) $${\hat{C}}_{\mathrm{cN}}^{*}=\frac{\mathrm{dk}}{b_{\max}-d}$$

and

(12) $${\hat{C}}_{\mathrm{aN}}^{*}=\frac{\mathrm{dk}}{\left(1-f\right)\left(b_{\max}-d\right)}.$$

Similarly, for the mutualist,

(13) $${\hat{C}}_{\mathrm{cM}}^{*}=\frac{\mathrm{dk}}{\left(b_{\max}\right)\left(1-s\right)-d}$$

and

(14) $${\hat{C}}_{\mathrm{aM}}^{*}=\frac{\mathrm{dk}}{\left(b_{\max}\right)\left(1-s\right)-d}.$$

**Figure 1:**
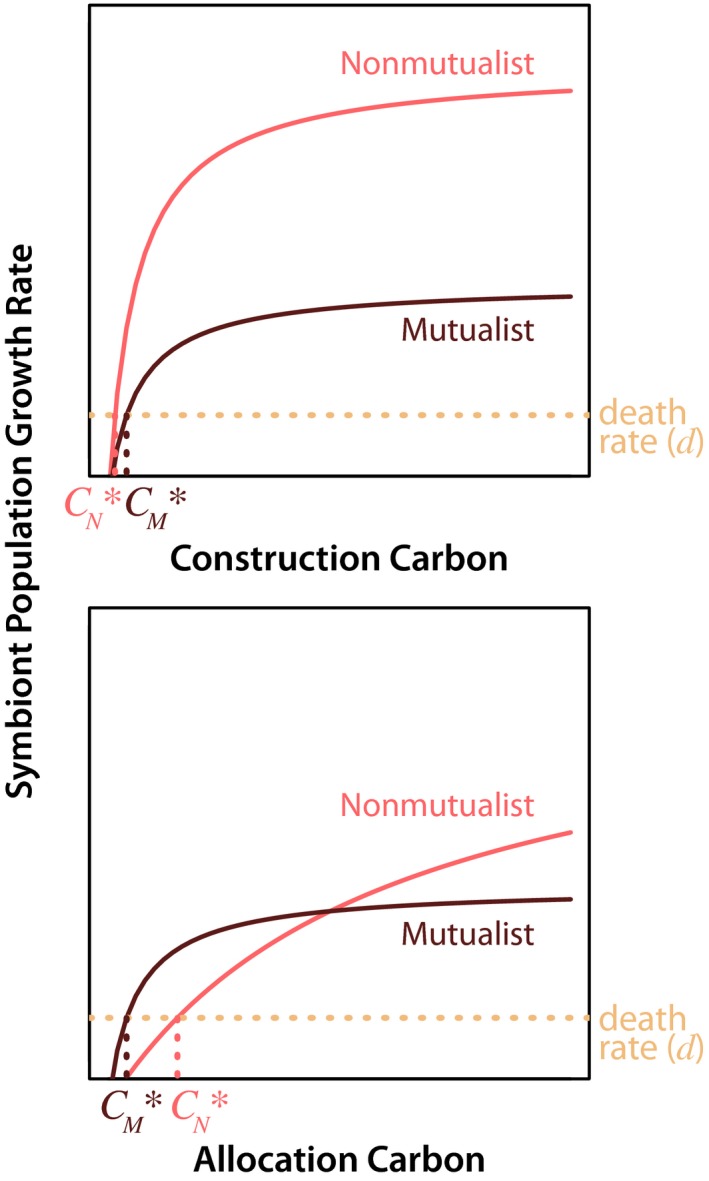
The nonmutualist outcompetes the mutualist for construction carbon (C_*N_ **< C*_M_) due to the cost of mutualism borne to the mutualist. The mutualist outcompetes the nonmutualist for preferentially allocated carbon (C_*M_ **< C*_N_), due to the fidelity of the plant to the mutualist

When we consider competition for construction carbon and allocation carbon in isolation from one another, we find that the nonmutualist outcompetes the mutualist for construction carbon (C_*N_ **< C_*M_ *)* due to the cost of mutualism borne to the mutualist (Figure 1). On the other hand, the mutualist outcompetes the nonmutualist for preferentially allocated carbon (C_*M_ **< C_*N_ *)* (Figure 1).

**Figure 2:**
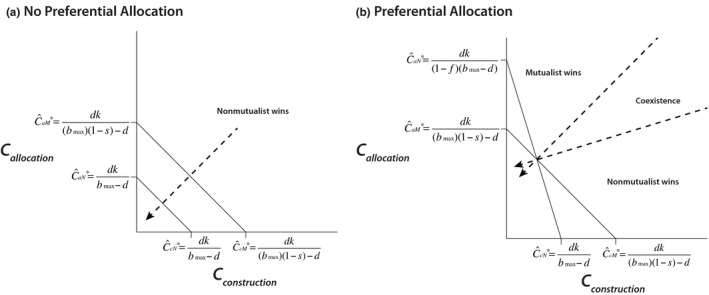
Competitive dynamics of the nonmutualist and mutualist competing for construction and allocation carbon. (a) In the absence of preferential allocation of carbon (*f *= 0), ${\hat{C}}_{\mathrm{aN}}^{*}={\hat{C}}_{\mathrm{cN}}^{*}$, resulting in a zero net growth isocline (ZNGI) with a slope of −1. Similarly, ${\hat{C}}_{\mathrm{aM}}^{*}={\hat{C}}_{\mathrm{cM}}^{*}$, which results in a ZNGI with a slope of −1, but which is higher than the nonmutualist ZNGI because of the cost of mutualism. In this scenario, the nonmutualist will always have competitive superiority. (b) In the presence of preferential allocation, ${\hat{C}}_{\mathrm{aN}}^{*}>{\hat{C}}_{\mathrm{cN}}^{*}$, creating a ZNGI for the nonmutualist with a slope of $\frac{-1}{\left(1-f\right)}$. The mutualist and nonmutualist ZNGIs cross such that C_*cN_ < C_*cM_ and C_*aM_ < C_*aN_, allowing for a condition for coexistence of mutualist and nonmutualist symbionts. Dotted lines represent consumption vectors, which are placed perpendicular to each ZNGI to indicate that allocated carbon and construction carbon are consumed in proportion to their availability

However, because these two separate carbon resources are substitutable, competitive outcomes are determined by the ratio in which the two resources are supplied. In the complete absence of preferential allocation (*f *= 0), ${\hat{C}}_{\mathrm{aN}}^{*}={\hat{C}}_{\mathrm{cN}}^{*}$ (see Equations (11) and (12)), and when ${\hat{C}}_{\mathrm{cN}}^{*}$ and ${\hat{C}}_{\mathrm{aN}}^{*}$ are plotted on two separate axes, a zero net growth isocline (ZNGI) is created with a slope of −1 (Figure 2a). Similarly, ${\hat{C}}_{\mathrm{aM}}^{*}={\hat{C}}_{\mathrm{cM}}^{*}$ (see Equations (13) and (14)), which results in a ZNGI with a slope of −1 (Figure 2a; see Appendix S1). These ZNGIs are parallel but not identical, because the cost of mutualism to the mutualist results in a higher ZNGI. Thus, in this scenario, the nonmutualist will always have competitive superiority.

However, in the presence of preferential allocation, ${\hat{C}}_{\mathrm{aN}}^{*}>{\hat{C}}_{\mathrm{cN}}^{*}$ (see Equations (11) and (12)), and when ${\hat{C}}_{\mathrm{cN}}^{*}$ and ${\hat{C}}_{\mathrm{aN}}^{*}$ are plotted on two separate axes, the ZNGI has a slope of $\frac{-1}{\left(1-f\right)}$ (see Appendix S1) which then intersects with the ZNGI for the mutualist (which still has a slope of −1; Figure 2b). Consumption vectors, which represent the change in resource availability due to consumption, were placed perpendicular to each ZNGI to indicate that allocated carbon and construction carbon are consumed in proportion to their availability to the mutualist and the nonmutualist, since symbionts cannot discriminate among sources of carbon. Thus, the mutualist depletes C_a_ and C_c_ equally, and the nonmutualist depletes C_c_ more than it does C_a_, due not to fundamental differences in the rate of consumption of these interchangeable carbon sources, but their availability to each symbiont (Figure 2b).

Thus, by examining Equations (5), (6) and (11)–(14), it can be inferred that if there is some cost of mutualism (s>0), the nonmutualist will outcompete the mutualist for construction carbon $\left(C_{\mathrm{cN}}^{*}<C_{\mathrm{cM}}^{*}\right)$. Furthermore, if degree of fidelity, $f>\frac{b_{\max}s}{b_{\max}-d}$, then the mutualist will outcompete the nonmutualist for allocated carbon $\left(C_{\mathrm{aM}}^{*}<C_{\mathrm{aN}}^{*}\right)$. These growth isoclines intersect in a manner that creates a condition for coexistence for the nonmutualist and mutualist (Figure 2b), whereby coexistence is possible when

(15) $$0<s\;\text{and}\;\frac{b_{\max}s}{b_{\max}-d}<f.$$

## 3 DISCUSSION

There is a longstanding paradox regarding how mutualistic interactions persist in the face of cheating, and a more recent restructuring of this paradox to ask how variation in partner quality persists in nature (Foster & Kokko, 2006; Heath & Stinchcombe, 2014). Our model demonstrates that resource competition coupled with preferential allocation may facilitate coexistence of plant root mutualists and nonmutualists, provided there are two carbon sources. Specifically, our model allows fidelity of the plant to the mutualist and the cost of mutualism to mediate competitive ability of the symbionts.

A central assumption of our model is that carbon constitutes two limiting resources. Our model has two nutritionally interchangeable, but temporally separated resource axes: carbon utilized for establishment of the symbiosis (e.g., construction of an arbuscule), and carbon preferentially allocated by the plant following symbiont establishment and assessment. In order for a plant to evaluate whether a symbiont is beneficial or nonbeneficial, the association must first be established and exchange must be initiated. As a result, the plant must pay a “start‐up cost” of carbon to the symbionts prior to the initiation of preferential allocation. Both the mutualist and the nonmutualist benefit from this carbon, which is distributed indiscriminately by the plant. Regardless of the benefit that the mutualist will eventually confer, the nonmutualist is a better competitor for this resource (i.e., has a lower C_*c_ than the mutualist) because it more efficiently converts this resource into its own growth. This advantage could be the simple consequence of not investing in costly resource exchange, but alternatively could also be due to secondary specialization on the indiscriminately allocated carbon, such as by increasing rates of dispersal or establishment to capture more of the plant's initial investment. Thus, in the absence of preferential allocation, the nonmutualist will always have a competitive advantage. On the other hand, by engaging in nutrient exchange with the plant, the mutualist is able to outcompete the nonmutualist for the carbon that is subsequently preferentially allocated by the plant. The mutualist will have a lower C_*a_ than the nonmutualist and thus is a better competitor on the allocated carbon axis. Therefore, in the presence of preferential allocation the two axes cross such that $\left({\hat{C}}_{N}^{*}<{\hat{C}}_{M}^{*}\right)$ for construction carbon, and $\left({\hat{C}}_{M}^{*}<{\hat{C}}_{N}^{*}\right)$ for allocated carbon, allowing for coexistence.

The carbon in this system originates solely from translocated photosynthate, but our model differentiates between carbon that is allocated first indiscriminately and then discriminately by the plant, such that time is an implicit assumption within the model. As such, these separate but substitutable carbon resources are temporal in nature, rather than compositional, which is consistent with recent experimental studies. Recent empirical research on preferential allocation by the plant host *Allium vineale* (wild onion) to mutualistic and nonmutualistic arbuscular mycorrhizal fungi demonstrated that in the presence of increasing soil phosphorous levels, the amount of carbon preferentially allocated to the mutualist decreased from ~35% of total allocated carbon to ~10%, whereas the amount of carbon allocated to the nonmutualist remained at a constant level of 10% of total allocated carbon (Ji & Bever, 2016). Using the same host plant, another study showed that a nonmutualist received a constant 11% of total allocated carbon, whereas a mutualist received 15%–25% of total carbon, depending on the amount of shade present (shading decreased preferential allocation to mutualists; Zheng et al., 2015). In these empirical examples, construction carbon would be included within the basal level of carbon allocated to both the nonmutualist and the mutualist, and the plant could then allocate additional carbon to the mutualist, depending on resource availability (e.g., phosphorous, light) and how reliant the plant was on the mutualist for nutrient uptake (Ji & Bever, 2016; Zheng et al., 2015). In the aforementioned greenhouse studies of *Allium vineale*, it appears that ~20% of the plant's carbon budget is used indiscriminately for root maintenance and construction of new arbuscules, half of which is captured by the nonmutualist and half by the mutualist. In general, it is likely that the availability of construction carbon would vary given the environment and the species involved in the interaction, but within a particular context, the carbon used for initial construction of symbioses would remain constant. Preferentially allocated carbon, however, would be more plastic depending on environmental conditions. Therefore, the amount of preferentially allocated carbon available to the mutualist could shift along its axis, resulting in three possible competitive outcomes at a given level of construction carbon (Figure 2b): (1) If preferentially allocated carbon were low, the nonmutualist would competitively exclude the mutualist; (2) after a threshold level of allocated carbon (determined by the resource axes) was reached, coexistence would be possible; and (3) if the degree of preferential allocation were sufficiently high, there would be competitive exclusion of the nonmutualist by the mutualist. Previous results demonstrate that preferentially allocated carbon decreases with increasing soil phosphorous (Ji & Bever, 2016) and with shade (Zheng et al., 2015) as might be expected from plant nutrition. Together, this predicts broad conditions for coexistence with environmental patterns of relative abundance of mutualists to nonmutualists.

By integrating resource competition between symbionts into our model, we derive a condition for mutualism that is more generous than that of Bever (2015). This is because the fidelity of the plant to the mutualist taxes the saturation of the nonmutualist per capita birth rate (Equation (1)). Biologically, this implies that due to host fidelity, the mutualist is able to achieve a higher birth rate at lower levels of carbon than expected in Bever (2015). Thus, even in the face of considerable and clearly documented costs to mutualists (Bronstein, 2001), fidelity of the plant to more mutualistic symbionts can act as a stabilizing mechanism for mutualism. The role of host fidelity in symbiont resource competition is supported by empirical studies. Greenhouse studies using arbuscular mycorrhizal fungi have shown that spatial structure of symbionts within plant root systems can facilitate the ease of partner choice by the plant such that mutualists receive more carbon and have a higher growth rate than nonmutualists (Bever et al., 2009). In nature, communities of arbuscular mycorrhizal fungi exhibit spatial structuring and clustering even at extremely small spatial scales (Davison et al., 2016; Horn, Caruso, Verbruggen, Rillig, & Hempel, 2014; Verbruggen et al., 2012). Additionally, in plant–rhizobia systems, selection should favor dishonest signaling of rhizobia quality (Heath & Tiffin, 2009), so plants do not know the efficacy of prospective rhizobial colonizers a priori. This means that nonmutualistic bacteria colonize alongside more cooperative bacteria (Denison & Kiers, 2011). Plants, however, may subsequently reward effective rhizobia (Batstone, Dutton, Wang, Yang, & Frederickson, 2017) or impose partner sanctions on ineffective bacteria to enforce mutualism (West, Kiers, Simms, & Denison, 2002) at multiple spatial scales (Kiers et al., 2003). However, according to our model, when the costs of mutualism are too high (see Equation (6)), mutualism is never a viable lifestyle even in the absence of competition with a nonmutualist. Thus, our model provides clear conditions for the coexistence of multiple root symbionts.

Even though our model shows that coexistence is possible within certain parameter space, these conditions alone do not necessarily explain why coexistence is so common in nature (Bever, Schultz, Pringle, & Morton, 2001). For example, in a boreo‐nemoral forest in Estonia, 10 plant species hosted a total of 47 arbuscular mycorrhizal taxa (Öpik, Metsis, Daniell, Zobel, & Moora, 2009). Even in an agricultural monoculture of sorghum, up to twelve species of arbuscular mycorrhizal fungi have been shown to coexist on a single crop (Schenck & Kinloch, 1980). However, our approach of coupling resource competition and preferential allocation, or in other words the concept that consumers compete for a good, and the supplier preferentially provides the good to the consumer that pays more, is compatible with the recent application of biological market theory to plantroot symbioses (Kiers et al., 2016; Kummel & Salant, 2006; Werner et al., 2014; Wyatt, Kiers, Gardner, & West, 2014). According to biological market theory, the volatility of biotic or abiotic conditions may drive the demand for specialized services and could even change which symbiont is considered the best quality partner in a system. Such variation would almost certainly alter preferential allocation of resources (Werner & Kiers, 2015), and a market that experiences frequent fluctuations would more likely maintain, on average, intermediate allocation levels. According to our model, intermediate levels of preferential allocation would lead to coexistence and the maintenance of variation in the mutualism. In contrast to biological market models, which only predict coexistence of symbionts under limited conditions (Kummel & Salant, 2006), our model easily explains both limitation of spread of nonbeneficial symbionts and their coexistence with mutualists by focusing on microbial competition. A level of preferential allocation low enough to competitively exclude the mutualist would potentially be biologically plausible if the resources being exchanged were freely available (Ji & Bever, 2016). This is consistent with the predictions of the negative physiological feedback model (Bever, 2015), which predicts that cheaters would potentially decline to zero with increasing resource availability. However, in many terrestrial ecosystems, nutrients such as nitrogen and phosphorous are limiting to the growth of plants (Vitousek, Porder, Houlton, & Chadwick, 2010). For instance, tropical soils are phosphorous‐limited and are also characterized by diverse communities of coexisting arbuscular mycorrhizal fungi (Janos, 1980). Moreover, plants could be selecting for coexistence of root symbionts (Moeller & Neubert, 2015), as associating with multiple mutualists may be beneficial for plant health and fitness. Increasing species richness of arbuscular mycorrhizal fungi has been shown to increase plant biodiversity, nutrient capture and productivity (van der Heijden et al., 1998), as well as plant growth (Koomen, Grace, & Hayman, 1987), although this effect may be due to increased likelihood of inclusion of particularly beneficial arbuscular mycorrhizal fungi (Vogelsang, Reynolds, & Bever, 2006).

Our model has been designed and interpreted primarily in the context of the specific interaction between plants and root symbionts, such as arbuscular mycorrhizal fungi and rhizobia, but could hold relevance for other mutualisms with a “pay‐first, evaluate‐later” dynamic. For instance, in mutualisms between plants and pollinating seed consumers (i.e., the yucca‐yucca moth and fig–fig wasp mutualisms), pollinators compete for host resources, but hosts can choose to allocate more resources to successfully pollinated fruits (in the case of figs, Jandér & Herre, 2016), or abort inadequately pollinated flowers (in the case of yucca, Huth & Pellmyr, 2000). In these systems, resources distributed to fruits or flowers before and after pollination would be analogous to our model's construction and allocation carbon terms, respectively. Previous research has shown that the intensity of sanctioning by fig hosts correlates negatively with the number of nonmutualists in a system (Jandér & Herre, 2010), which supports our theoretical finding that when host fidelity is high and the cost of mutualism is low, competing mutualists and nonmutualists can coexist. Conversely, this model may be less relevant to ant–plant mutualisms, in which plants provide domatia (structures that ants can live inside) and extrafloral nectaries or food bodies. In return, ants defend the plant from herbivores or competitors and thus promote the growth of large plants, which can then provide more resources to the resident ant colony, increasing ant colony growth (Frederickson & Gordon, 2009). Because only a single ant colony typically resides on a single plant, this positive feedback between plant and ant colony growth makes cheating an inviable strategy for either partner (Jones et al., 2015) and nullifies the need to pay first and then evaluate. Our model may also be irrelevant to the squid‐*Vibrio* mutualism, which relies on a rigorous progression of colonization occurring in a series of stages, in which each step confers greater specificity between the host and the symbiont (Nyholm & McFall‐Ngai, 2004). This “obstacle course” effectively excludes *Vibrio* cheaters and cosmopolitan bacteria from colonizing (Leigh, 2010).

Overall, we demonstrate a condition for root symbiont coexistence when symbionts compete for plant carbon, and plants preferentially allocate some of that carbon to mutualists. We treated carbon as two nutritionally interchangeable, but temporally separated resources—carbon indiscriminately allocated to initial construction and evaluation of the symbiosis, and carbon preferentially allocated to the best mutualist after symbiosis establishment. Our results are consistent with previous empirical studies as well as an emerging literature on the application of biological market theory to plantroot symbioses. This model does not consider processes such as physiological feedbacks between partners and hosts, which could expand the conditions for coexistence of symbionts varying in benefit. Moreover, our model treats mutualists and nonmutualists as binary and does not account for symbiont variation along the mutualism–parasitism spectrum. Under a more realistic model, it may be easier to predict how colonization by a community of symbiotic partners or various selection pressures may alter mutualistic strategies (Wyatt et al., 2014). While empirical work is necessary to test basic assumptions and predictions, our model adds a new dimension of biological complexity, resource competition, to previous theoretical work, and strengthens our understanding of the maintenance of variation in mutualism.

## CONFLICT OF INTEREST

None declared.

## AUTHOR CONTRIBUTIONS

N.C. and J.D.B. developed the model. N.C. wrote the first draft of the article and J.D.B. provided substantial revisions. Both authors approved the final version. Neither author has a conflict of interest.
