# Supplementary material for: Carbon allocation and competition maintain variation in plant root mutualisms
Source: Ecol Evol. 2018 May 4;8(11):5792–800. doi: 10.1002/ece3.4118 (PMC6010867; doi:10.1002/ece3.4118)
Supplement: Supplementary file 1 [file ECE3-8-5792-s001.docx]

**Intermediate steps: SOLVING FOR CROSSING PER CAPITA BIRTH RATES**

We derive the point at which mutualist and non-mutualist have equal R-star values by first setting mutualist and non-mutualist birth functions (Equations 1 and 2) equal to each other and solving for the C value at this point.

$$\frac{b_{\text{max}}\left( 1-f \right)C}{k+\left( 1-f \right)C}=\frac{b_{\text{max}}\left( 1-s \right)C}{k+C}$$

$$\left( {k+C)(b}_{\text{max}}\left( 1-f \right)C \right)=\left( k+\left( 1-f \right)C \right)\left( b_{\text{max}}\left( 1-s \right)C \right)$$

$$0=\left( k+\left( 1-f \right)C \right)\left( b_{\text{max}}\left( 1-s \right)C \right)-(\left( {k+C)(b}_{\text{max}}\left( 1-f \right)C \right))$$

$$0=\left( k+C-Cf \right)\left( b_{\text{max}}\left( C-Cs \right) \right)-(k+C)(b_{\text{max}}(C-Cf)$$

$${0=(k+C-Cf)(b}_{\text{max}}C-b_{\text{max}}Cs)-(k+C)(b_{\text{max}}C-b_{\text{max}}Cf)$$

$${0=(kb}_{\text{max}}C-kb_{\text{max}}Cs+C^{2}b_{\text{max}}-C^{2}b_{\text{max}}s-C^{2}b_{\text{max}}f+C^{2}b_{\text{max}}fs)-(kb_{\text{max}}C-kb_{\text{max}}Cf+C^{2}b_{\text{max}}-C^{2}b_{\text{max}}f)$$

$$0=-kb_{\text{max}}Cs-C^{2}b_{\text{max}}s+C^{2}b_{\text{max}}fs+kb_{\text{max}}Cf$$

$$0=b_{\text{max}}(-ksC-sC^{2}+C^{2}fs+kCf)$$

$$0=-ksC-sC^{2}+C^{2}fs+kCf$$

$$0=C(-ks-sC+Cfs+kf)$$

$$0=-ks-sC+Cfs+kf$$

$$Cfs-sC=ks-kf$$

$$C(fs-s)=k(s-f)$$

$$C=\frac{k(s-f)}{fs-s}$$

$C=\frac{k(s-f)}{s(f-1)}$ (Eq. 3)

We then plug Equation 3 into either one of the per capita birth rate equations (Equations 1 or 2), to solve for the birth rate for the mutualist and the non-mutualist at their point of intersection. Here we demonstrate this using *W_M_*, but the solution would be the same for *W_N_.*

$W_{M}=\frac{b_{\text{max}}(1-s)\frac{k(s-f)}{s(f-1)}}{k+\frac{k(s-f)}{s(f-1)}}$

$$W_{M}=\frac{b_{\text{max}}(1-s)\frac{k(s-f)}{s(f-1)}}{\frac{k(s(f-1)}{s(f-1)}+\frac{k(s-f)}{s(f-1)}}$$

$$W_{M}=\frac{b_{\text{max}}(1-s)\frac{k(s-f)}{s(f-1)}}{\frac{k\left( sf-s \right)+ks+kf}{s(f-1)}}$$

$$W_{M}=\frac{b_{\text{max}}(1-s)\frac{k(s-f)}{s(f-1)}}{\frac{ksf-ks+ks+kf}{s(f-1)}}$$

$$W_{M}=\frac{\frac{b_{\text{max}}(1-s)k(s-f)}{s(f-1)}}{\frac{fk(s-1)}{s(f-1)}}$$

$$W_{M}=\frac{b_{\text{max}}(1-s)k(s-f)}{fk(s-1)}$$

$$W_{M}=\frac{b_{\text{max}}(1-s)(s-f)}{f(s-1)}$$

$$W_{M}=\frac{b_{\text{max}}(-1)(s-1)(s-f)}{f(s-1)}$$

$$W_{M}=\frac{b_{\text{max}}(-1)(s-f)}{f}$$

$$W_{M}=\frac{b_{\text{max}}(f-s)}{f}$$

$W_{M}=\frac{b_{\text{max}}\left( f-s \right)}{f}=W_{N}$ (Eq. 4)

If the death rate, *d*, is greater than this value, the non-mutualist has a lower *C** and would exclude the mutualist regardless of the level of plant preferential allocation. If *d* is less than this value, mutualism is possible. We derive this condition by setting this value greater than *d* and solving for *f*.

$$W_{M}=\frac{b_{\text{max}}\left( f-s \right)}{f}>d$$

$$\frac{b_{\text{max}}\left( f-s \right)}{f}>d$$

$$\frac{b_{\text{max}}\left( f-s \right)}{f}-d>0$$

$$\frac{b_{\text{max}}\left( f-s \right)}{f}-\frac{fd}{f}>0$$

$$b_{\text{max}}\left( f-s \right)-fd>0$$

$$b_{\text{max}}f-b_{\text{max}}s-fd>0$$

$${f(b}_{\text{max}}-d)-b_{\text{max}}s>0$$

$${f(b}_{\text{max}}-d)>b_{\text{max}}s$$

$f>\frac{b_{\text{max}}s}{b_{\text{max}}-d}$ (Eq. 5)

Provided $b_{\text{max}}>d$ for any fungi to grow, then when $s=0$ (no cost of mutualism), *f* must be greater than 0. And when $s=1$ (total cost of mutualism), *f* must be greater than $\frac{b_{\text{max}}s}{b_{\text{max}}-d}$. However, because this number is greater than 1, stability of the mutualism is impossible. Because *f* has a maximum of 1, then $\frac{b_{\text{max}}s}{b_{\text{max}}-d}$ must be less than 1 for mutualism to be possible. Thus, solving for *s,* there is a condition whereby the mutualist has a competitive advantage when

$s<\frac{b_{\text{max}}-d}{b_{\text{max}}}$ (Eq. 6)

**Intermediate steps: NON-MUTUALIST COMPETITIVE ABILITY FOR BOTH RESOURCE AXES**

We start by setting the birth rate of the non-mutualist equal to 0 to solve for critical level of carbon (*C**) that maintains the symbiont birth rate above the constant death rate, *d*.

$W_{N}=\frac{b_{\text{max}}C_{N}}{k+C_{N}}-d$ (Eq. 7)

$0=\frac{b_{\text{max}}C_{N}}{k+C_{N}}-d$

$$\frac{b_{\text{max}}C_{N}}{k+C_{N}}=d$$

$$b_{\text{max}}C_{N}=d\left( k+C_{N} \right)$$

$$b_{\text{max}}C_{N}=dk+dC_{N}$$

$$b_{\text{max}}C_{N}-dC_{N}=dk$$

$$C_{N}(b_{\text{max}}-d)=dk$$

$\hat{C}_{N}^{*}=\frac{dk}{b_{\text{max}}-d}$ (Eq. 9)

Because $C_{N}=\left( 1-f \right)C_{a}{+C}_{c}$

And because $C_{a}$ and $C_{c}$ are completely interchangeable, if $C_{a}=0$, (meaning 100% of carbon is $C_{c}$), then

$\hat{C}_{cN}^{*}=\frac{dk}{b_{\text{max}}-d}$ (Eq. 11)

And if $C_{c}=0$, (meaning 100% of carbon is $C_{a}$), then

$$C_{aN}\left( 1-f \right)=\frac{dk}{b_{\text{max}}-d}$$

$\hat{C}_{aN}^{*}=\frac{dk}{{(1-f)(b}_{\text{max}}-d)}$ (Eq. 12)

To solve for slope of the ZNGI,

Slope = $-\frac{\hat{C}_{aN}^{*}}{\hat{C}_{cN}^{*}}$ = $-\frac{\frac{dk}{{(1-f)(b}_{\text{max}}-d)}}{\frac{dk}{b_{\text{max}}-d}}$ = $-\frac{1}{1-f}$

**Intermediate steps: MUTUALIST COMPETITIVE ABILITY FOR BOTH RESOURCE AXES**

We start by setting the birth rate of the mutualist equal to 0 to solve for critical level of carbon (*C**) that maintains the symbiont birth rate above the constant death rate, *d*.

$W_{M}=\frac{b_{\text{max}}{(1-s)C}_{M}}{k+C_{M}}-d$ (Eq. 8)

$0=\frac{b_{\text{max}}{(1-s)C}_{M}}{k+C_{M}}-d$

$$\frac{b_{\text{max}}{(1-s)C}_{M}}{k+C_{M}}=d$$

$$b_{\text{max}}{(1-s)C}_{M}=d(k+C_{M})$$

$$b_{\text{max}}{(1-s)C}_{M}=dk+dC_{M}$$

$$b_{\text{max}}{(1-s)C}_{M}-dC_{M}=dk$$

$${C_{M}(b}_{\text{max}}(1-s)-d=dk$$

$\hat{C}_{M}^{*}=\frac{dk}{(b_{\text{max}})(1-s)-d}$ (Eq. 10)

Because $C_{M}=C_{c}+C_{a}$

And because $C_{a}$ and $C_{c}$ are completely interchangeable, if $C_{a}=0$, (meaning 100% of carbon is $C_{c}$), then

$\hat{C}_{cM}^{*}=\frac{dk}{{(b}_{\text{max}})(1-s)-d}$ (Eq. 13)

And if $C_{c}=0$, (meaning 100% of carbon is $C_{a}$), then

$\hat{C}_{aM}^{*}=\frac{dk}{{(b}_{\text{max}})\left( 1-s \right)-d}$ (Eq. 14)

To solve for slope of ZNGI,

Slope = $-\frac{\hat{C}_{aM}^{*}}{\hat{C}_{cM}^{*}}$ = $-\frac{\frac{dk}{{(b}_{\text{max}})\left( 1-s \right)-d}}{\frac{dk}{{(b}_{\text{max}})\left( 1-s \right)-d}}$ = $-1$
